# Preventive and Therapeutic Role of Functional Ingredients of Barley Grass for Chronic Diseases in Human Beings

**DOI:** 10.1155/2018/3232080

**Published:** 2018-04-04

**Authors:** Yawen Zeng, Xiaoying Pu, Jiazhen Yang, Juan Du, Xiaomeng Yang, Xia Li, Ling Li, Yan Zhou, Tao Yang

**Affiliations:** ^1^Biotechnology and Germplasm Resources Institute, Yunnan Academy of Agricultural Sciences, Agricultural Biotechnology Key Laboratory of Yunnan Province, Kunming 650205, China; ^2^Kunming Tiankang Science & Technology Limited Company, Kunming Yunnan 650231, China; ^3^Biomedical Engineering Research Center, Kunming Medical University, Kunming 650500, China; ^4^Clinical Nutrition Department, The Second People's Hospital of Yunnan Province, Kunming Yunnan 650021, China

## Abstract

Barley grass powder is the best functional food that provides nutrition and eliminates toxins from cells in human beings; however, its functional ingredients have played an important role as health benefit. In order to better cognize the preventive and therapeutic role of barley grass for chronic diseases, we carried out the systematic strategies for functional ingredients of barley grass, based on the comprehensive databases, especially the PubMed, Baidu, ISI Web of Science, and CNKI, between 2008 and 2017. Barley grass is rich in functional ingredients, such as gamma-aminobutyric acid (GABA), flavonoids, saponarin, lutonarin, superoxide dismutase (SOD), K, Ca, Se, tryptophan, chlorophyll, vitamins (A, B1, C, and E), dietary fiber, polysaccharide, alkaloid, metallothioneins, and polyphenols. Barley grass promotes sleep; has antidiabetic effect; regulates blood pressure; enhances immunity; protects liver; has anti-acne/detoxifying and antidepressant effects; improves gastrointestinal function; has anticancer, anti-inflammatory, antioxidant, hypolipidemic, and antigout effects; reduces hyperuricemia; prevents hypoxia, cardiovascular diseases, fatigue, and constipation; alleviates atopic dermatitis; is a calcium supplement; improves cognition; and so on. These results support that barley grass may be one of the best functional foods for preventive chronic diseases and the best raw material of modern diet structure in promoting the development of large health industry and further reveal that GABA, flavonoids, SOD, K-Ca, vitamins, and tryptophan mechanism of barley grass have preventive and therapeutic role for chronic diseases. This paper can be used as a scientific evidence for developing functional foods and novel drugs for barley grass for preventive chronic diseases.

## 1. Introduction

Barley (*Hordeum vulgare* L.) is the fourth most important cereal crop in the world and has the highest dietary fiber content; its malt for functional food is not only the world's largest material for beer, but also often used as one of 300 species being used in Chinese herbal medicine. Regular consumption of whole grain barley and its hydroalcoholic extract reduces the risk of chronic diseases (diabetes, cancer, obesity, cardiovascular disease, etc.), based on phytochemicals including *β*-glucan, phenolic acids, flavonoids, lignans, tocols, phytosterols, and folate [1, 2]. Barley with preventive inflammatory and cardiovascular diseases has exhibited activities against all human platelet agonists inhibited both cyclooxygenase and lipoxygenase pathways of arachidonic acid metabolism, which elevated the SOD and GSH-Px activities [3].

Barley with cold and frost tolerance of growing at 4000 m is a key for ancient Tibetans climb to 3400 m [4]; Tibetan Plateau is an important origin and domestication base of cultivated barley [5]. Human *Flt3* ligand from barley is a glycoprotein including *α*(1,3)-fucose and *α*(1,2)-xylose, which showed expression of human growth factor in barley grains with active protein [6]. The amino acid concentration in barley grass irradiated by artificial light (red 9 + blue 1) is greater than that by natural light, which can increase *γ*-tocopherol by 100% red light [7], but cyanogenic glucosides content is 4% less than that by sunlight [8]. The accumulation of lutonarin (isoorientin-7-*O*-glucoside) and 3-feruloylquinic acid (C_17_H_20_O_9_) and xanthophyll-cycle pigments is greatly increased by high photosynthetically active radiation and ultraviolet exposure in barley leaves [9]. Chronic disease of human beings is associated with the five evolutionary stages of the major dietary guidelines (i.e., the healthiest major dietary guidelines for modern humans): fruits or vegetables, grass or Cyperaceou, cereals (rice, wheat, millet, beans, barley, and corn), polished rice or wheat flour, and white rice or wheat flour + grass powder [10].

Barley grass (BG) has young green leaves and stem of vegetative growth stage from seedling at 10 days after sprouting (barley sprout) to elongation stage (barley green) for nutritional peak before the start of reproductive cycle of barley [11–13]; however, *Vrs2* is associated with floral architecture by regulating hormonal homeostasis and gradients in barley [14]. BG is not only consumed as a popular green-colored drink [15], but also used in preventive chronic diseases, especially circulatory disorders, anticancer, reducing obesity, antidiabetes, anti-arthritis, reducing cholesterol, antioxidant, and anti-inflammation [12]. Light can promote cytokinin degradation and the formation of bioactive cytokinins in barley leaves, which has a positive correlation between cytokinin oxidase/dehydrogenase activity and senescence in most cases [16]. The amino acid and vitamin C content in hydroponic BG are higher than those in organic soil [17]. In spray-dried barley grass powder with good solubility and small size, its contents of the chlorophyll, flavonoids, and SOD enzyme activity are 56.7%, 68.1%, and 47.9% of vacuum freeze-dried powder with high nutrition and good color, respectively [18]. Although BG has played an important role in human health, coevolution and functional ingredients as well as major mechanism in therapeutic role between preventive chronic diseases and young barley grass for functional foods of human beings are unclear.

## 2. Functional Ingredients of Barley Grass

Barley grass is rich in nutritious and functional ingredients, in which major ingredients content according to dried barely grass include dietary fiber 29.5%, protein 27.3%, fat 4.57%, vitamin A 20.5 mg/100 g, vitamin C 251.6 mg/100 g, Ca 479.4 mg/100 g, S 305.5 mg/100 g, Cr 0.14 mg/100 g, Fe 23.3 mg/100 g, Mg 183.2 mg/100 g, K 3384 mg/100 g, chlorophyll 528.5 mg/100 g, SOD 440.0 U/g, catalase 839 U/g, lutonarin 342.9 mg/100 g, saponarin 726.2 mg/100 g, total flavonoid 0.53%, total polyphenol 1.06%, ABTS (RC50) 53.3 *μ*g/mL, GABA 150.5 mg/100 g, and tryptophan 810.0 mg/100 g (see Table 1). Generally, the content of nutritious and functional ingredients is very different depending on the growth stage of barley grass or processing technology or various cultivars; for example, the sodium content in mountainous region is low but high in saline and alkaline land as well as vegetable land, and the content of dietary fiber at seedling stage is low but high at elongation stage. There are greater differences of saponarin and lutonarin contents in barley leaves at the growth stage; in particular, its lutonarin content at shooting period is 6.4 times higher than that at one leaf period, and its saponarin content in two leaves at one period is 6.5 times higher than that at heading period [11]. There are greater differences of tryptophan contents in barley leaves under three light sources [7]. Many studies have shown that BG contains significant quantities of Ca, Fe, Zn, K, Mg, folic acid, *β*-carotene, chlorophyll, pantothenic acid, vitamin C, and vitamin B12 [12]. Mean contents of chlorophyll (SPAD value), soluble solids, betaine, and flavonoid in BG of 100 cultivars are 44.53, 70.39 mg/g fresh weigh (FW), 2333.99 *μ*g/g FW, and 4114.25 *μ*g/g FW, respectively [25]. BG contains 30 times thiamine (C_12_H_16_N_4_OS) and 11 times Ca than that of cow's milk, 6.5 times carotene and 5 times Fe content of spinach, 7 times vitamin C (C_6_H_8_O_6_) in oranges, 4 times thiamine in whole wheat flour [12, 26], 2 times protein in barley grains [27], its total flavonoids and alkaloids are 2.1 times, 10.7 times, and GABA 37.8 times of brown rice [10].

### 2.1. GABA and Amino Acids

Gamma-aminobutyric acid is an inhibitory neurotransmitter that reduces neural excitability in the mammalian central nervous system with three subclasses of receptors, namely, relaxing, antianxiety, and anticonvulsive; alleviates pain; regulates sleep; and increases cognitive and reproductive effects [28]. GABA (C_4_H_9_NO_2_), glutamic acid (C_5_H_9_NO_4_), and CaCl_2_ play significant roles in alleviating cold-induced effects by restoration of membrane integrity [29]. Barley bran is more efficient than wheat bran in the GABA production [30]. GABA contents of BG for Fudamai 1 and Fan 11 are 143~183 mg/100 g and 125~151 mg/100 g, respectively [24]. GABA can alleviate oxidative damage of H^+^ and Al^3+^ toxicities in BG by activating antioxidant defense and reducing the carbonylated proteins [31]. BG contains 20 amino acids with energy production, cell building, and regeneration, especially 8 essential amino acids [12, 19].

### 2.2. Flavonoids

Higher dietary flavonoid intake associated with gastric cancer risk decreased in European population [32]. The microbiome contributes to diminished postdieting flavonoid levels and ameliorates excessive secondary weight gain [33]. Barley green contains total flavonoids of 1.12% and DPPH free radicals scavenging potential of 78.52%; however, betaine and total flavonoids can be kept at room temperature, but soluble protein and soluble total sugar and SOD could be better kept in cold storage [34]. The total flavonoid contents in BG increased from 273.1 to 515.3 CE mg/100 g between 13 and 56 days after sprouting; however, lutonarin (isoorientin-7-*O*-glucoside) has stronger radical scavenging activity than saponarin (isovitexin-7-*O*-glucoside); its antioxidant ability is improved with growth time, which exhibited high total polyphenol (44.37–55.07%) [13]. Total flavonoid extraction in BG is 94.66 mg/100 g [35]. There are 37 flavonoids, and hydroxycinnamates in BG include saponarin (C_27_H_30_O_15_), lutonarin, isoorientin (C_21_H_20_O_11_), isoscoparine (C_22_H_21_O_11_), *C*-glycosyl flavones, *O*-glycosyl-*C*-glycosyl flavones, *O*-diglycosyl flavones, isoscoparin-7-*O*-glucoside derivatives, 7-*O*-[6-acyl]-glucoside, and -7-*O*-[6-acyl]-glc-4′-glucoside of isovitexin [36]. The major flavonoids from BG extract are isovitexin-7-*O*-glucoside (54.17%) and isoorientin-7-*O*-glucoside (33.36%) [37]. The major flavonoid antioxidants in BG are the flavone-*C*-glycosides, saponarin, and lutonarin [38]. Lutonarin and saponarin account for 71–75% of ten phenolics for BG, which contain 24.0 mg/100 g lutonarin and 14.0 mg/100 g saponarin [23, 39]. BG from Syrian contains the derivatives of flavonols, quercetin (C_15_H_10_O_7_), and isorhamnetin (C_16_H_12_O_7_), but flavonoids with glycosylation and acylation as well as hydroxycinnamate glycosides, esters, and amides in methanolic extracts from different regions of the world [40].

### 2.3. Enzymes

BG contains 300 enzymes of body utilization, such as superoxide dismutase (SOD), catalase (CAT), guaiacol peroxidase (POD), ascorbate peroxidase (APX, cellular imaging), aspartate aminotransferase (association with vitamin B6), cytochrome oxidase and hexokinase (association with mitochondria), deoxyribonuclease, fatty acid oxidase, malic dehydrogenase (allosteric regulation), nitrate reductase, RNase, P4D1, nitrogen oxyreductase, peroxidase, peroxidase catalase, phosphatase, phospholipase, polyphenol oxidase, transhydrogenase, and glycosyl isovitexin; but enzymes are not found in cooked foods [41, 42]. l-Phenylalanine ammonia lyase is the first enzyme in the biosynthesis of phenylpropanoid-derived plant compounds such as flavonoids, coumarins, and the cell wall polymer lignin [43]. Antioxidant enzymes in BG include SOD, CAT, POD, APX, lipid peroxidation, protein oxidation, DNase activity, and DNA damage, which are oxidative biomarkers in response to Al^3+^ stress [44]. SOD has powerful anti-inflammatory activity; CAT is one antioxidant enzyme, which may provide resistance against many diseases, such as cancer [45]. The erythrocyte zinc and SOD activity are influenced by metabolic syndrome, plasmatic glucose, body mass index, and waist circumference [46]. Mean of SOD activity in barley leaves is 4.11 ± 1.31 U/mg [47]. Zn^2+^, Cu^2+^, and Mn^2+^ can inhibit significant CAT and SOD at higher contents, but Cd^2+^, Hg^2+^, and Pb^2+^ significantly restrain CAT and SOD at different contents in BG [48]. H_2_S is a signaling molecule in plants and animals; H_2_S treatment maintains higher POD activity in gibberellic acid-treated layers and higher SOD, POD, CAT, and APX activities in non-GA-treated barley aleurone layers [49].

### 2.4. Minerals

An increase in K^+^ intake is a major nutritional approach in preventing hypertension, heart, and Alzheimer's disease as well as improving cognitive performance by decreasing inflammation and oxidative stress [10, 50]. Chronic kidney disease can cause cardiovascular disease and mortality, which is related with vascular calcification and abnormal electrolytes; however, hypocalcemia can cause mortality in patients with heart failure [51]. Due to their sulfide and quercetin mechanism in the treatment of chronic diseases, garlic and onion have anticancer properties; prevent cardiovascular and heart diseases; have anti-inflammatory properties; reduce obesity; have antidiabetic, antioxidant, antimicrobial, neuroprotective, and immunological effects; and so on [52]. BG contains the highest mineral content, especially potassium, calcium, iron, and sulfur (see Table 1); its K is 14.3 times, Ca 33.2 times, Fe 13.4 times, sulfur 3.3 times of brown rice [53]. The *qK1*/*qMg1*/*qCa1* region between markers Bmag0211 and GBMS0014 on chromosome 1H is shown to have large additive effects for Mg, Ca, and K concentrations in grains [54].

### 2.5. Chlorophyll

Chlorophyll and heme are the fundamental pigments of life. The biosynthetic pathways of methane production include chlorophyll, heme, and vitamin B12 [55]. The chlorophyll and soluble protein content in BG decreased with increasing seeding rate [22]. Photosystem II core dimers began to dissociate monomers at 40–50°C for heated (1°C/min) barley leaves, and chlorophyll-containing protein complexes appeared at 57–60°C [56]. The rate of CO_2_ fixation and chlorophyll contents decreased, but flavonoids and carotenoids as well as enzyme activity increased, when etiolated barley seedlings at UV-B irradiation (312 nm) for 5 h [57]. Chlorophyll has anti-inflammatory and antioxidant properties and reduces fecal, urinary, and body odor [12]. Chlorophyll derivatives may play a significant role in anticancer activity, because it exhibits similar antimutagenic effect to 3-methylcholanthrene [58]. Chlorophyll content in barley grass is 542.9 mg/100 g (see Table 1); its extraction yield is 1364.6 mg/100 g [59]. The chlorophyll and total flavonoids content in barley grass under optimum combined drying conditions are 600.6 ± 19.2 and 569.5 ± 14.5 mg/100 g, respectively [17].

### 2.6. Vitamins

Fruit and vegetable (400 g/day) are associated with higher blood vitamin contents, especially antioxidant and B vitamins [60]. Diabetes is an oxidative inflammatory stress disease; however, it is necessary to monitor their vitamin B12 contents [61]. High vitamin C is used as homeostasis of brain-resident microglia [62]. Vitamin D deficiency is prevalent worldwide, which can prevent diabetes, cancer, depression, and so on [63]. Vitamins can treat nonalcoholic steatohepatitis (V_E_) and chronic hepatitis C virus (V_B12_ and V_D_), reduce gallstones (V_C_), aphthous stomatitis (V_B12_), and inflammatory bowel disease (V_D_ and V_B1_), and so on [64]. The vitamin content (vitamin C, 0.52%, vitamin E, 73.06 mg/kg) of BG in Sebastian is higher than that in Malz (0.50%, 61.84 mg/kg) and AF Lucius (0.51%, 6.78 mg/kg) [65]. BG includes vitamin A 20.5 mg/100 g, V_B1_ 0.61 mg/100 g, V_B2_ 1.56 mg/100 g, V_B6_ 1.12 mg/100 g, V_E_ 15.0 mg/100 g, and V_C_ 251.6 mg/100 g (see Table 1).

### 2.7. Polyphenols

500 polyphenols are distributed across a wide variety of foods; a protective role of dietary polyphenols against chronic diseases includes preventing cardiovascular diseases, diabetes, and cancer [66], due to their antioxidant and anti-inflammatory properties, and improving blood pressure and lipids as well as insulin resistance, which may reduce the risk of all-cause mortality [67]. The total polyphenol contents in BG increased from 776.6 to 1060.1 GAE mg/100 g between 13 and 40 days after sprouting, but decreased at day 56 to 982.6 GAE mg/100 g, in which it has higher antioxidant activity at 40 days after sprouting; ABTS^+^ scavenging assay, the RC50 values of BG, decreased from 111.0 to 53.3 *μ*g/mL between 13 and 40 days then increased to 55.3 *μ*g/mL on day 56 [13]. The total phenolic content is 26.55 mg/100 g on 23 days after the sowing, but 13.91 mg/100 g on 56 days for BG, which are major hydroxycinnamic acid, orientin, isoorientin, and isovitexin derivatives [39].

## 3. Preventive Chronic Disease of Barley Grass

BG has the complete abundant nutrition including chlorophyll, superoxide dismutase, lutonarin, saponarin, vitamins, minerals, and eight essential amino acids [12, 68], but heat will destroy lots of nutritional values. Barley sprouts with saponarin showed anti-inflammatory and antioxidant activities; BG possess lots of health effects which include antioxidant, hypolipidemic, antidepressant, and antidiabetes [69]. Its manufacture is organically barley leaves squeezing, juice of low temperature, spray-dried in 3 seconds to stabilize grass powder [12]. BG has lots of health effect, such as hypolipidemic, hypoglycemic, preventive constipation, and anticancer, antioxidant, and anti-inflammatory activities [12]. Daily consumption of barley grass powder promotes sleep; regulates blood sugar and pressure; enhances immunity and liver function; detoxifies acne skin; improves gastrointestinal function; prevents constipation; has anticancer and anti-inflammatory effects; alleviates atopic dermatitis; loses weight and hypolipidemic; reduces gout and hyperuricemia; prevents heart disease; has bone injury recovery, lustihood, and anti-fatigue effects; repairs memory; has antiaging effect; and so on [10].

### 3.1. Promote Sleep and Functional Ingredients of Barley Grass

Barley grass powder with higher GABA, Ca, K, and tryptophan contents is a very effective functional food in promoting sleep [70]. Sleep symptoms are associated with intake of specific dietary nutrients including Ca (OR = 0.83) and K (OR = 0.70) [71]. Barley grass powder for Yungong brand contains 62 times more GABA and 99 times more Ca as well as 31 times more K than that for polished rice [70]. The effective foods of improving sleep for modern people are polished rice or wheat flour plus BG powder and its products [70].

### 3.2. Antidiabetes and Functional Ingredients of Barley Grass

BG and its extract can scavenge oxygen free radicals, improve health, based on protective vascular diseases, and impair the pancreas endocrine of diabetic patients [72, 73]; its dietary fiber has a significant reduction in fasting blood sugar and blood glucose [74]. Saponarin in BG can control the postprandial blood glucose of diabetes [75]. Barley grass powder (1.2 g/day) within two months can significantly reduce fasting blood sugar, glycated hemoglobin, total cholesterol, and low-density lipoprotein (LDL) cholesterol, but significantly increase the high-density lipoprotein (HDL) cholesterol levels [76]. Hexacosanol in barley leaf can improve cholesterol metabolism by decreasing cholesterol synthesis [77]. Adenosine 5′-monophosphate-activated protein kinase in barley sprouts can regulate cell glucose that is a target for drugs against diabetes and obesity; however, policosanol content of 10 days after sprouting (3.437 g/kg) is 3 times higher than that of 5 days (1.097 g/kg) [78]. Polyamines in barley cells can increase under stress conditions, and it has a similar insulin function and antiglycating effect; however, the raising circulation of polyamines stop the glycation reaction under hyperglycemic concentrations [79].

### 3.3. Regulating Blood Pressure and Functional Ingredients of Barley Grass

The analogy porphyrin heads between chlorophyll (Mg) and hemoglobin (Fe) have an important therapeutic effect for chlorophyll in hemoglobin deficiency [12]. Chlorophyll and heme biosynthesis are regulated to adapt environment and plant development; 5-aminolevulinic acid provides for tetrapyrrole synthesis (Mg and Fe), however rapid dark inhibition of 5-aminolevulinic acid (C_5_H_9_NO_3_) synthesis in BG [80]. Saponarin is a flavonoid found in BG that possesses potent regulating blood pressure [81]. Barley grass helps blood flow and digestion as well as general toxification of human's body, which related to superoxide dismutase and lutonarin as well as saponarin [12]. Barley grass powder with lowering hypertension has higher minerals (K and Ca) and GABA as well as lower Na [82]; its K, Ca, and GABA contents at autumn sowing under cold and high altitude (2010 m) are 3110, 845, and 377.46 mg/100 g, respectively [70]. Total free amino acid concentration varied very smaller, but the greater change from glutamate to GABA in BG and induced GABA gene expressions under cold acclimation and frost tolerance [83].

### 3.4. Enhance Immunity and Functional Ingredients of Barley Grass

Structural complexity of arabinoxylan of polysaccharide can be responsible for the immunomodulatory activity in young barley leaves [84]; high altitude cultivars (1200~3500 m) showed higher arabinoxylan (39.8–68.6%), anthocyanin (11.0–60.9%), *β*-glucan (7.5–30.8%), and metal chelating activity (16.6–43.2%) than plains (97–126 m) [85]. *β*-1,3-1,4-Glucan is a major accumulating component in cell wall of BG [86]. Glucuronoarabinoxylan and rhamnogalacturonan-I polysaccharide branched with arabinogalactan II side chain with immunostimulatory can be important for expression given the association with macrophage stimulatory activity in barley leaf [87].

### 3.5. Protective Liver and Functional Ingredients of Barley Grass

Barley sprouts with abundant saponarin possess the liver-protective effect by inhibiting the inflammatory response induced by alcohol [69]. Saponarin showed hepatoprotection and antioxidation against liver damage of CCl_4_ in vitro and in vivo [88]. SOD enzymes are separated into three types of genes such as SOD1 (CuZn-SOD), SOD2 (Mn-SOD), SOD3 (extracellular SOD) in mammals. SOD1 (CuZn-SOD) deficiency can cause universal free radical damage and developing liver cancer in life [89].

### 3.6. Beauty Anti-Acne/Detox and Functional Ingredients of Barley Grass

Some exciting research has found that BG has the strongest ability to degrade six organophosphate pesticides; however, degradation of six pesticides (10 mg/L) in a 15% solution of young green barley leaves for 3 h at 37°C and pH 7.4 is malathion (100%, C_10_H_19_O_6_PS_2_) = chlorpyrifos (100%, C_9_H_11_Cl_3_NO_3_PS) > parathion (75%, C_10_H_14_NO_5_PS) > diazinon (54%, C_12_H_21_N_2_O_3_PS) > guthion (41%, C_10_H_12_N_3_O_3_PS) > methidathion (23%, C_6_H_11_N_2_O_4_PS_3_) [90]. Barley metallothioneins (MTs) have difference in intracellular homeostasis of metal ions specifically Cu detoxification, most MTs are downregulated by more Zn or Cd, and expression of MT1a, MT2b1, MT2b2, and MT3 in barley leaves increased more than 50 times during 10 d after germination [91].

### 3.7. Antidepressant and Functional Ingredients of Barley Grass

Depression not only is one of the most widely associated mental disorders in the world, but also has been associated with the risk to develop cancer, dementia, obesity, diabetes, blood pressure, atherosclerosis, epilepsy, and stroke. There is an important role for GABAergic, glutamatergic, and cholinergic receptors in the pathomechanism of depression [92, 93]. The reduce epilepsy and preventive dementia [10] as well as antidepressant of the young green barley leaf are regulated by inhibiting the hippocampus levels increased of nerve growth factor [94]. The vitamins and minerals in BG can prevent the stress-related psychiatric disorders of depression [95].

### 3.8. Improve Gastrointestinal Function and Functional Ingredients of Barley Grass

Young barley leaf powder with the water-insoluble dietary fiber can increase the fecal volume and laxative action by stimulating gut tract by the pH lowering [15]. BG is very effective in the treatment of ulcerative colitis [96], pancreatitis, and disorders of the gastrointestinal tract [15]. A germinated barley foodstuff is helpful to reduce ulcerative colitis and improves symptoms by promoting the probiotics growth [97]. Selenium-enriched barley grass has significant ameliorative effect on ethanol-induced gastric ulcer in mice [98].

### 3.9. Anticancer and Functional Ingredients of Barley Grass

BG inhibits the cancer cell growth by the combined effects of high alkaline, strong antioxidative, phytochemicals, flavonoids, and chlorophyll [99, 100]. There is a very good antitumor effect for the phytochemical mixtures of BG in breast cancer [100]. BG can be served as health food for dialysis patients based on its absorbed exogenous functional ingredients applied from the outside [101]. Green barley extract has anticancer effect by its antiproliferative and proapoptotic functions on leukemia and lymphoma as well as breast cancer cells of human beings [102]. BG tricin can inhibits melanin production in melanoma cells, based on a hydroxyl group at the C-4′ position and methoxy groups at the C-3′,5′ positions of the tricin skeleton [103]. Yunnan province has the lowest cancer mortality and is the largest producer of anticancer barley, but Shanghai in China has the highest cancer mortality, which is associated with the sharp decline of barley [104].

### 3.10. Anti-Inflammation and Functional Ingredients of Barley Grass

BG has anti-inflammatory properties and heals the intestinal lining, which is used for gastrointestinal tract disorders, pancreatitis, recovering illness, and the treatment of ulcerative colitis [15, 96, 105]. Saponarin from barley sprouts is a very important functional ingredient of natural anti-inflammation [106]. Barley grass extract is very effective in the treatment of rheumatoid arthritis than that of SOD consumption [107, 108], which may be served as a supplement in the treatment of urologic and gynaecologic disorders as well as airway infections [12]. BG extract with antioxidant and anti-inflammation can be used as natural drug for the treatment of patients with rheumatoid arthritis by scavenge ROS and downregulate TNF-*α* production from peripheral blood and synovial fluid of patients [108]; however, green barley juice is part of arthritis therapy [109].

### 3.11. Antioxidants and Functional Ingredients of Barley Grass

Natural antioxidants in plant major include polyphenols, flavonoids, vitamins, and volatile chemicals [110]. Barley is among the most stress-tolerant crops, its flag leaf *γ*-tocopherol, glutathione and succinate content by same genes encoding enzymes of the pathways producing antioxidant metabolites [111]. The antioxidant phytonutrients of barley grass include the superoxide dismutase, 2^″^-*O*-glucosyl isovitexin (2^″^-*O*-GIV), and protoheme [112–114]. Flavonoids (lutonarin and saponarin) with antioxidative effects have been isolated from young barley [99, 115], in which lutonarin and saponarin contents in barley grass increase with UV exposure [36]. Saponarin in barley grass possesses strong antioxidant activities, which can prevent diseases caused by oxidative damage such as various cancers, inflammations, and cardiovascular diseases [81]. Isoorientin and orientin possessed potent antioxidant effects with IC_50_ values of 20.765 ± 651.1 and 27.565 ± 657.36508 M (DPPH) and 5.765 ± 650.3 and 8.265 ± 650.36508 M (ABTS), respectively [39]. Barley leaves extracted by methanol and ethanol may be alternatives to synthetic antioxidants in the food industry [114]. Barley leaf powder can be incorporated into raw minced pork as natural additives to retard oxidation [116]. Feeds supplemented with barley leaves containing antioxidants enhanced pork quality by increasing the levels of unsaturated oleic and gondoic acids [117].

### 3.12. Hypolipidemic and Functional Ingredients of Barley Grass

Barley green can modulate lipid metabolism, resist lipid peroxidation, improve vascular endothelium, and prevent atherosclerosis [118]. This 30% inhibition of hyperlipidemic atherosclerosis by barley leaf is associated with a decrease in plasma lipids and an increase in antioxidative abilities [73]. 2^″^-*O*-Glycosyl isovitexin from BG is more effective than *α*-tocopherol towards fatty acid esters at higher levels [119]. Barley sprout contains 4.97% fat, 52.6% polysaccharide, 34.1% protein, vitamins, minerals, and polyphenols, which show significant lipid-lowering [77].

### 3.13. Antigout/Hyperuricemia and Functional Ingredients of Barley Grass

Barley grass reduces blood uric acid, which has lots of benefits on feces metabolism, lipids metabolism, liver function, and antioxidant system for human [120]. A fermented barley extract can reduce uric acid effect on hyperuricemia [121]; however, SOD and alkaloid are focused on the treatment of arthritis, bursitis, and gout [122, 123]. A fermented barley extract P reduces serum uric acid by increasing its urinary excretion [124].

### 3.14. Preventive Cardiovascular Diseases and Functional Ingredients of Barley Grass

BG antioxidation may contribute to the prevention of cancer and metabolic disorders as well as cardiovascular diseases [115]. BG can prevent thrombosis and cardiovascular diseases by enhancing better blood viscosity and flow [125]. Normal tryptophan metabolism of barley grass is a developing appropriate therapies for the symptoms of cardiovascular disease patients [126].

### 3.15. Antihypoxia/Anti-Fatigue and Functional Ingredients of Barley Grass

BG is rich in flavones that have antihypoxia and anti-fatigue effects on humans, especially the total contents of lutonarin and saponarin amounting to 17.0% [127]. The barley seedling (1 g/mL) has significant effect on anti-fatigue in mice, especially the exhausting swimming and antianoxic time with significant longer, which reduced blood glucose significantly of diabetes inducted by alloxan (C_4_H_4_N_2_O_5_) and gastric ulcer induced by alcohol [128].

### 3.16. Preventive Constipation and Functional Ingredients of Barley Grass

Young barley leaf powder has lots of health effects, such as preventive constipation [129]. The dietary fiber of germinated barley alleviates constipation via the proliferation of the colonic crypts in loperamide (C_29_H_33_ClN_2_O_2_)-administered rats [130]; however, dietary fiber content of BG is 29.5% (see Table 1).

### 3.17. Alleviated Atopic Dermatitis and Functional Ingredients of Barley Grass

GABA_B_ receptor is a new therapeutic way to treat inflammatory skin diseases [131]. Combined administration of fermented barley extract P and GABA alleviated atopic dermatitis by regulating the Th1/Th2 balance to a Th1-immune response [132]. GABA (377.46 mg/100 g) of Yungong BG is 62.5 times and 37.8 times of polished and brown rice [10, 70].

### 3.18. Preventive Heart Disease and Functional Ingredients of Barley Grass

Western countries have more incident of coronary heart disease than that of stroke and diabetes for Asian countries, based on the loss of K and Mg as well as dietary fiber of major food from whole wheat to wheat flour [10]. An increase in K^+^ intake can prevent heart disease which associate with decreasing inflammation and oxidative stress [10, 50]. K (3110 mg/100 g) of Yungong BG is 31 times of polished rice [10, 70].

### 3.19. Calcium Supplement and Functional Ingredients of Barley Grass

Calcium homeostasis is paramount physiological and pathophysiological importance in health and disease [133]. BG can be used as the prevention or treatment of osteoporosis [134]. Yungong BG has the health effect due to the highest Ca content (845 mg/100 g) that is 99.6 times of polished rice [10, 70].

### 3.20. Improve Cognition and Functional Ingredients of Barley Grass

GABA and K have increasing cognitive effects [10, 28, 50] due to higher concentration, such that GABA (377.46 mg/100 g) and K (3110 mg/100 g) of Yungong BG is 62.5 times and 31 times of polished rice [10, 70].

### 3.21. Preventive Other Diseases and Functional Ingredients of Barley Grass

BG of Yungong brand has also lustihood, bone injury recovery, antiaging, losing weight, reducing blood fat [10]. Carotene is a fat-soluble vitamin, which plays a very important role in the health of the retina, lungs, gastrointestinal tract, brain, and immune system [135]. Vitamin B1 (daily intake 2.0 mg) is a water-soluble vitamin, which has a favorable impact on the digestive, cardiovascular, and nervous systems [136].

## 4. Major Mechanisms of Functional Ingredients of Barley Grass for Preventive Chronic Disease

### 4.1. GABA Mechanism for Preventive Chronic Diseases

GABA (C_4_H_9_NO_2_) in BG promotes sleep, is antidiabetic, regulates blood pressure, enhances immunity, protects liver, is antidepressant, improves gastrointestinal function, is anti-inflammatory and antioxidant, prevents cardiovascular and heart diseases, alleviates atopic dermatitis, increases cognition, and so on (Table 2). Sleep is regulated by neurotransmitter systems of GABA and dopamine signaling, which improves the sleeping quality [152]. GABA has a very important intraislet transmitter in adjusting islet-cell secretion and anti-inflammatory and immunoregulatory activities, which can treat diabetes by promoting the regenerative functions and against apoptosis of *β*-cell [138]. GABA can inhibit an increase in blood pressure and accelerate growth hormone secretion, however GABA associated with CO_2_ concentrations [141]. Baclofen (C_10_H_12_ClNO_2_) of GABA_B_ receptor agonist regulates Toll-like receptor 3 and Toll-like receptor 4 signaling in glia and immune cells, which has the therapeutic role in neuroinflammatory disease [142]. Hepatic encephalopathy is related with a regional reduce GABA levels in the visual cortex due to liver failure and cerebral osmolytic disorders [143]. Antidepressant effect of ascorbic acid and ketamine in tail suspension test may involve an activation of GABA_A_ receptors and a possible inhibition of GABA_B_ receptors [144]. GABA and GABA receptor function can modulate gastrointestinal motility and inflammation [145]. Antioxidants L-carnitine (C_7_H_15_NO_3_) and d-methionine (C_5_H_11_NO_2_S) regulate cortical electrical spike activity through GABA_A_ receptor activation [147]; GABA_B_ receptor positive allosteric modulators are very important in the treatment of alcoholism [153]. GABA can treat cardiovascular diseases that is associated with glycoprotein VI in platelet membrane, such as stroke and myocardial infarction, in which GABA inhibited platelet activation stimulated by convulsed and prolonged the closure time of whole blood and the occlusion time of platelet plug formation [148, 149]. The central mechanisms are an interaction between angiotensin II receptor 1 and interleukin-1 beta with decrease GABA expression in the paraventricular nucleus contributing to progression in heart failure [150]. Bone marrow mesenchymal stem cell transplantation can improve cognitive impairment via upregulating the hippocampal GABAergic system in a rat model of chronic cerebral hypoperfusion [154].

### 4.2. Flavonoids Mechanism for Preventive Chronic Diseases

Flavonoids especially saponarin and lutonarin in BG have antidiabetic effect; regulate blood pressure; protect liver; have antidepressant, anticancer, anti-inflammatory, antioxidant, and hypolipidemic effects; prevent cardiovascular diseases; have antihypoxia and anti-fatigue effects; and so on (Table 2). Saponarin from young barley leaves can regulate gluconeogenesis and glucose uptake by activated AMPK in a calcium-dependent manner [155]. Saponarin and lutonarin with regulating blood pressure in BG can promote blood flow, digestion, and detoxification of whole body [12]. The liver-protective effect of saponarin is blocked nitric oxide synthase and cyclooxygenase expression; however, antidepressant of saponarin in BG is associated with its anti-inflammation and antioxidation [69]. Anti-inflammation of saponarin in barley is LPS-induced macrophages via inhibition of NF-*κ*B, ERK, and p38 signaling [106]. Saponarin/lutonarin (4.5/1) from BG has very strong antioxidation using cod liver oil, *ω*-3 fatty acids, phospholipids, and blood plasma [156]. Twenty-five secondary metabolites (saponarin, lutonarin, etc.) are putatively identified in healthy and diseased barley leaves, which play a role in adaptation to unfavorable growth conditions [157].

### 4.3. SOD Mechanism for Preventive Chronic Diseases

SOD in BG has antidiabetic, anti-inflammatory, antioxidant, antigout and hyperuricemia, and anticancer effects, prevents cardiovascular disease, alleviates atopic dermatitis, improves cognition, and so on (Table 2). Oxygen radical absorbance capacity values are associated with the increases in CAT and SOD activities and the reduction in reactive oxygen species, in which the extract has a significant therapeutic and preventive potentials of cancer and diabetes [139]. Zinc is cofactor of SOD with antioxidant defense in type 2 diabetes by regulating the glutathione metabolism and metallothionein expression, competing with Fe and Cu in the cell membrane, inhibiting nicotinamide adenine dinucleotide phosphate-oxidase enzyme (C_21_H_29_N_7_O_17_P_3_) [158]. Gout patients have oxidative stress and HDL protective effects against atherosclerosis dependent on paraoxonase-1 activity, which correlated positively with SOD, negatively with malondialdehyde, and oxidized LDL [159]. SOD is a characteristic of cardiovascular alterations in hypertension and diabetes, which is associated with alterations in vascular structure and function [160]. Atopic dermatitis patients can be damaged by oxidants, which is evident from an increase of malondialdehyde and a decrease of SOD and CAT enzymatic antioxidants [149]. Hippocampal-dependent cognitive functions have the aid of production of new neurons and dendritic structures; however, oxidative stress plays an important part in the SOD-deficient and radiation environment, which can be effective use of SOD deficiency in cognitive functions and identify therapeutic methods [151]. The natural ROS-scavenging compounds for vitamins and SOD of barley grass are crucial and promising therapeutic strategies for vascular repair [161].

### 4.4. K-Ca Mechanism for Preventive Chronic Diseases

K or Ca in BG promotes sleep, has antidiabetic effect, regulates blood pressure, prevents cardiovascular and heart diseases, is a calcium supplement, increases cognition, and so on (Table 2). Large-conductance Ca^2+^-activated K^+^ channels regulate rhythmicity for sleep-wake in suprachiasmatic nucleus neuronal actions [162]. The role of Ca^2+^-activated K^+^ channels for endothelial cell in uterine vascular dysfunction of diabetes, especially the impaired function of IKCa channels [163]. Small conductance Ca^2+^-activated K^+^ channels not only expressed in the paraventricular nucleus play a key role in the regulation of arterial blood pressure and cardiovascular function [140], but also inhibited lots of situations of atrial fibrillation in the heart under normal and pathophysiological conditions [164]. Barley seedling extracts inhibited RANKL-induced osteoclast differentiation with alteration of I*κ*B degradation, c-Fos, and NFATc1 molecules in osteoclastogenesis [134].

### 4.5. Vitamins Mechanism for Preventive Chronic Diseases

Vitamins in BG promote sleep; have antidepressant, antioxidant, hypolipidemic, gastrointestinal, and anti-inflammatory effects; prevent cardiovascular diseases; and so on (Table 2). Vitamin B1 has a favorable impact on the sleep and gastrointestinal and cardiovascular systems, which stimulates the brain and improves the psycho-emotional state [136]. V_C_ treatment can prevent the spatial memory impairment of chronic sleep deprivation by the antioxidant defense mechanisms of the hippocampus [137]. Vitamins in BG have very effective antistress capabilities by preventing reduction in the wheel-running activity and hippocampal mRNA of brain-derived neurotrophic factor in response to restraint stress [95]. The vitamin E has unique therapy chronic diseases, antioxidation and anti-inflammations, based on scavenge active nitrogen, inhibit cyclooxygenase- and 5-lipoxygenase-catalyzed eicosanoids, as well as suppress proinflammatory signaling [165]. Antioxidants vitamins C and E in BG may prevent cardiovascular diseases, which decrease total cholesterol, LDL cholesterol, and oxygen free radicals [72].

### 4.6. Tryptophan Mechanism for Preventive Chronic Diseases

Tryptophan in BG promotes sleep, has anti-inflammatory effect, prevents cardiovascular diseases, and so on (Table 2). Tryptophan may improve sleep quality in stress-vulnerable individuals carrying the 5-hydroxytryptamine transporter-linked promoter region S-allele [166]. Kynurenine and tryptophan as well as their ratio will contribute to the interplay between inflammation, metabolic syndrome, mood disturbance, anemia, and cardiovascular diseases [146]. The kynurenine pathway of tryptophan degradation in preventive cardiovascular diseases is very important by an inducible indoleamine 2,3-dioxygenase-mediated tryptophan metabolism [126]. Lineage-specific duplications of genes associated in the transport of nutrients to developing seeds and the mobilization of carbohydrates in barley grains [167].

## 5. Conclusion Remarks and Future Perspectives

The data summarized in current review point out that major mechanism and more than 30 functional ingredients of barley grass exert potent preventive exceed 20 chronic diseases. The result reveals coevolution between preventive chronic diseases and young barley grass for functional foods of human beings. We can suggest that chronic diseases of human beings are associated with six major dietary structures: (i) fruits/vegetables, (ii) young grass/barley grass, (iii) carnivorous, (iv) cereal crops (rice, wheat, millet, beans, barley, and corn), (v) polished rice or wheat flour, and (vi) white rice or wheat flour + grass powder. The modern diet of polished rice and wheat flour is the key for the outbreak of chronic diseases in human beings, but white rice (wheat flour) + barley grass powder is the healthiest major dietary guidelines for modern humans. Although functional ingredients of barley grass for preventing and treating chronic diseases seem a complicated task, the barley grass powder is the best functional foods of cell nutrition and detoxification in marketable nutritional and health products all over the world, which is associated with coevolution for the similar center of origin (Africa, especially Ethiopia) between human beings and barley, because a health product can prevent and cure more than 20 chronic diseases which has not been reported, which may still open new venues for therapeutic interventions. Regular consumption of barley has 8000 years history, which may become a successful and safe strategy to treat chronic disease conditions. These data support that barley grass powder is rich in GABA, flavonoids, SOD, K-Ca, vitamins, and tryptophan, which are known to play a pivotal role in many chronic diseases.

This review provides important information and effective strategies that will guide future research and production of functional foods, especially GABA, flavonoids, SOD, K-Ca, vitamins, and tryptophan for prevention and treatment of chronic diseases. BG of Yungong brand can prevent more than 20 chronic diseases, which is associated with its rich functional components, based on its growth stage from autumn via winter to summer, drying process under sunlight at 1900–2300 m at spring with dry and windy conditions; Yunnan province at 1900–2300 m can plant barley in the four seasons (spring, summer, autumn, and winter) and may be associated with the rich and complex enzyme system in BG. Further researches are indispensable to resolve lots of problems, such as a better understanding of the interconnection between other 20 functional ingredients and preventing chronic diseases in clinical trials as well as ecological contribution. Further studies are necessary to unravel major pathological mechanism of coevolution between preventive chronic diseases and young barley grass for functional foods of human beings. This review may be used as a starting point for novel nutraceuticals, functional foods, or complementary and alternative drugs to maintain or improve the chronic diseases in barley grass.

## Figures and Tables

**Table 1:** Functional and nutrient compositions of dried barley grass.

Composition contents	Mean ± SD	Range	*N*	References
Calories (KJ/100 g)	1333 ± 315	1055~1607	4	[12, 19]
Sodium (mg/100 g)	328.2 ± 288.4	50~833	6	[12, 19]
Carbohydrates (%)	57.9 ± 5.1	55.2~64.0	4	[12, 19]
Dietary fiber (%)	29.5 ± 15.5	2.0~39.1	5	[12, 19]
Protein (%)	27.3 ± 4.3	19.9~34.1	7	[12, 19, 20]
Fat (%)	4.57 ± 1.31	2.30~6.03	6	[12, 19, 20]
Vitamin A (mg/100 g)	20.5 ± 4.7	14.4~25.0	4	[12, 19]
Vitamin B1 (mg/100 g)	0.61 ± 0.40	0.05~1.19	5	[12, 19, 20]
Vitamin B2 (mg/100 g)	1.56 ± 0.65	0.24~2.41	5	[12, 19, 20]
Vitamin B3 (mg/100 g)	7.18 ± 7.39	2.20~16.49	4	[12, 19]
Vitamin B6 (mg/100 g)	1.12 ± 0.97	0.18~2.58	5	[12, 19]
Vitamin B12 (mg/100 g)	1.16 ± 0.26	0.81~1.40	4	[12, 19, 20]
Vitamin C (mg/100 g)	251.6 ± 239.1	19.4~548.0	5	[19]
Vitamin E (mg/100 g)	15.0 ± 14.1	6.1~46.1	4	[19, 20]
Calcium (Ca, mg/100 g)	479.4 ± 172.5	330~819	6	[19–21]
Phosphorus (P, mg/100 g)	380.4 ± 60.7	283~430	5	[19, 21]
Chromium (Cr, mg/100 g)	0.14 ± 0.06	0.09~0.21	3	[19]
Copper (Cu, mg/100 g)	1.66 ± 1.25	0.72~3.50	6	[19–21]
Sulfur (S, mg/100 g)	305.5 ± 6.4	301~310	2	[19]
Iron (Fe, mg/100 g)	23.3 ± 10.1	10.0~41.2	6	[19–21]
Magnesium (Mg, mg/100 g)	183.2 ± 46.0	110~247.1	7	[19, 21]
Manganese (Mn, mg/100 g)	3.94 ± 1.56	2.3~5.4	5	[19, 21]
Molybdenum (Mo, mg/100 g)	0.048 ± 0.006	0.042~0.053	3	[19]
Potassium (K, mg/100 g)	3384 ± 649	2400~4300	7	[19, 21]
Zinc (Zn, mg/100 g)	3.43 ± 1.36	1.80~5.68	6	[19, 21]
Chlorophyll (mg/100 g)	542.9 ± 88.2	438~662	5	[17, 19, 22]
Superoxide dismutase (U/g)	440.0 ± 39.8	416~1382	4	[19]
Catalase (U/g)	839.0 ± 142.7	675~935	3	[19]
Lutonarin (mg/100 g)	342.9 ± 92.3	200.0~540.0	21	[11, 23]
Saponarin (mg/100 g)	726.2 ± 250.1	300.0~1260.0	21	[11, 23]
Total polyphenol (%)	1.06 ± 0.02	1.03~1.08	4	[13]
Total flavonoid (mg/100 g)	526.2 ± 52.7	487.5~593.4	5	[13, 17]
ABTS (RC50, *μ*g/mL)	53.3 ± 9.1	41.8~63.8	4	[13]
GABA (mg/100 g)	150.5 ± 24.2	125~183	4	[24]
Tryptophan (mg/100 g)	736.7 ± 569.0	290~1400	6	[7]

**Table 2:** Functional ingredients of barley grass for therapeutic chronic disease.

Number	Chronic disease	Functional ingredients	References
1	Promote sleep	GABA, Ca, K, tryptophan, vitamin C	[70, 71, 137]
2	Antidiabetes	Saponarin; dietary fiber, Ca; AMP-activated protein kinase, polyamines; GABA; SOD	[74, 75, 78, 79, 138, 139]
3	Regulating blood pressure	Saponarin; lutonarin, K, Ca; GABA	[12, 81, 82, 140, 141]
4	Enhance immunity	Arabinoxylan; polysaccharide; GABA	[84, 87, 142]
5	Protective liver	Saponarin; SOD; GABA	[69, 88, 89, 143]
6	Beauty anti-acne/detox	Metallothioneins	[91]
7	Antidepressant	GABA; saponarin; vitamins and minerals	[10, 69, 93, 95, 144]
8	Improve gastrointestinal	Dietary fiber; selenium; GABA	[15, 98, 145]
9	Anticancer	Alkaline, flavonoids, chlorophyll; tricin; SOD	[99, 100, 103, 139]
10	Anti-inflammation	Chlorophyll; saponarin; SOD; GABA; tryptophan	[12, 106–108, 145, 146]
11	Antioxidants	Chlorophyll; lutonarin, saponarin; isoorientin and orientin; *γ*-tocopherol, glutathione; SOD, flavonoid, protein P4D1; GABA	[12, 39, 81, 111–114, 147]
12	Hypolipidemic	Saponarin; *α*-tocopherol; 2^″^-*O*-glycosyl isovitexin, polysaccharide	[69, 77, 119]
13	Antigout/hyperuricemia	Alkaloid, SOD	[122, 123]
14	Preventive cardiovascular diseases	Saponarin; tryptophan; vitamins (A, B1, C, E), SOD; K, Ca; GABA	[72, 81, 87, 126, 136, 140, 148]
15	Antihypoxia/anti-fatigue	Flavones (lutonarin, saponarin)	[127]
16	Preventive constipation	Dietary fiber	[130]
17	Alleviated atopic dermatitis	GABA, SOD	[131, 132, 149]
18	Preventive heart disease	K, GABA	[10, 50, 150]
19	Calcium supplement	Ca	[10, 50, 70, 134]
20	Improve cognition	GABA, K, SOD	[10, 28, 50, 151]

## References

[1] Idehen E., Tang Y., Sang S. (2017). Bioactive phytochemicals in barley. *Journal of Food and Drug Analysis*.

[2] Minaiyan M., Ghannadi A., Movahedian A., Hakim-Elahi I. (2014). Effect of *Hordeum vulgare* L. (barley) on blood glucose levels of normal and STZ-induced diabetic rats. *Research in Pharmaceutical Sciences*.

[3] Gul S., Ahmed S., Kifli N. (2014). Multiple pathways are responsible for anti-inflammatory and cardiovascular activities of *Hordeum vulgare* L. *Journal of Translational Medicine*.

[4] Chen F. H., Dong G. H., Zhang D. J. (2015). Agriculture facilitated permanent human occupation of the Tibetan plateau after 3600 B.P. *Science*.

[5] Wang Y., Ren X., Sun D., Sun G. (2016). Molecular evidence of RNA polymerase II gene reveals the origin of worldwide cultivated barley. *Scientific Reports*.

[6] Erlendsson L. S., Muench M. O., Hellman U. (2010). Barley as a green factory for the production of functional *Flt3* ligand. *Biotechnology Journal*.

[7] Koga R., Meng T., Nakamura E. (2013). The effect of photo-irradiation on the growth and ingredient composition of young green barley (Hordeum vulgare). *Agricultural Sciences*.

[8] Meng T., Nakamura E., Irino N. (2015). Effects of irradiation with light of different photon densities on the growth of young green barley plants. *Agricultural Sciences*.

[9] Klem K., Holub P., Štroch M. (2015). Ultraviolet and photosynthetically active radiation can both induce photoprotective capacity allowing barley to overcome high radiation stress. *Plant Physiology and Biochemistry*.

[10] Zuo Y., Zeng Y., Pu X. Strategies of functional foods for heart disease prevention in human beings.

[11] Jia J., Li Y. L., Shen Y. H., Du Y. Z. (2010). Accumulation of lutonarin and saponarin in barley leaves during growth and differences in their contents among different varieties. *Food Science*.

[12] Lahouar L., El-Bok S., Achour L. (2015). Therapeutic potential of young green barley leaves in prevention and treatment of chronic diseases: an overview. *The American Journal of Chinese Medicine*.

[13] Park M. J., Seo W. D., Kang Y.-H. (2015). The antioxidant properties of four Korean barley cultivars at different harvest times and profiling of major metabolites. *Journal of Agricultural Science*.

[14] Youssef H. M., Eggert K., Koppolu R. (2017). VRS2 regulates hormone-mediated inflorescence patterning in barley. *Nature Genetics*.

[15] Ikeguchi M., Tsubata M., Takano A. (2014). Effects of young barley leaf powder on gastrointestinal functions in rats and its efficacy-related physicochemical properties. *Evidence-based Complementary and Alternative Medicine*.

[16] Schlüter T., Leide J., Conrad K. (2011). Light promotes an increase of cytokinin oxidase/dehydrogenase activity during senescence of barley leaf segments. *Journal of Plant Physiology*.

[17] Meng F., Xv Y., Hu J., He D., Jiang J. (2017). Optimization of combined drying process by hot-air and microwave for barley seedling powder. *Journal of Food Safety and Quality*.

[18] Gao T., Zhang M., Han Y., Huang S. B. (2015). Effect of two drying methods on the quality of barley grass powder. *Journal of Food and Biotechnology*.

[19] Jiazhen Y. A., Yawen Z. E., Xiaomeng Y. A., Xiaoying P. U., Juan D. U. (2016). Utilization of barley functional foods for preventing chronic diseases in China. *Agricultural Science & Technology*.

[20] Duan Q.-H., Li Y., Ge Z.-X. (2014). Analysis and evaluation of nutritional components of barley leaves powder. *Chinese Medicines Journal of Research and Practice*.

[21] Zeng Y.-W., Wang L.-X., Yang X.-M. (2016). Difference of elements in different types of seedling powder and Its grains of barley recombinant inbred lines. *Scientia Agricultura Sinica*.

[22] Chen J.-F., Zhang Y., Zhang Q.-Y. (2014). Effect of sowing amount on yield and quality of barley seedlin. *Fujian Journal of Agricultural Sciences*.

[23] Chen T., Wang P., Du Y., Shen Y., Li Y. (2012). Preparative isolation and purification of lutonarin and saponarin from barley seedlings by HSCCC. *Journal of Liquid Chromatography & Related Technologies*.

[24] Zhang Y., Liao C.-J., Xiao W.-H., Lan X.-L., Zhang Q.-Y., Chen J.-F. (2017). Impact of different cutting period on barley seedling production, rationing and *γ*-aminobutyric acid content. *Journal of Triticeae Crops*.

[25] Xia Y. S., Ning Z. X., Li R. H., Guo P. G. (2012). Analysis on bioactive substances of tender leaves in different barley cultivars. *Science and Technology of Food Industry*.

[26] Hagiwara Y., Hagiwara H., Ueyama H. (2001). Physiologically active substances in young green barley leaf extract. *Nippon Shokuhin Kagaku Kogaku Kaishi*.

[27] Xin P. Y., Pu X. Y., Du J., Yang T., Zeng Y. W. (2016). Protein content determination of barley grain and seedling powder. *Journal of Triticeae Crop*.

[28] Manayi A., Nabavi S. M., Daglia M., Jafari S. (2016). Natural terpenoids as a promising source for modulation of GABAergic system and treatment of neurological diseases. *Pharmacological Reports*.

[29] Jia Y., Zou D., Wang J. (2017). Effects of *γ*-Aminobutyric Acid, Glutamic Acid, and Calcium Chloride on Rice (Oryza sativa L.) Under Cold Stress During the Early Vegetative Stage. *Journal of Plant Growth Regulation*.

[30] Jin W. J., Kim M. J., Kim K. S. (2013). Utilization of barley or wheat bran to bioconvert glutamate to *γ*-aminobutyric acid (GABA). *Journal of Food Science*.

[31] Song H., Xu X., Wang H., Wang H., Tao Y. (2010). Exogenous gamma-aminobutyric acid alleviates oxidative damage caused by aluminium and proton stresses on barley seedlings. *Journal of the Science of Food and Agriculture*.

[32] Bo Y., Sun J., Wang M., Ding J., Lu Q., Yuan L. (2016). Dietary flavonoid intake and the risk of digestive tract cancers: a systematic review and meta-analysis. *Scientific Reports*.

[33] Thaiss C. A., Itav S., Rothschild D. (2016). Persistent microbiome alterations modulate the rate of post-dieting weight regain. *Nature*.

[34] Xia Y. S., Ning Z. X., Li R. H., Guo P. G. (2012). Study on optimization of extraction technology and stability during storage of barley green. *Science and Technology of Food Industry*.

[35] Zhang H., Qiao Y. J., Qi W. Y. (2013). Optimization of extraction process of total flavonoids from young barley leaves. *Food and Fermentation Industries*.

[36] Ferreres F., Andrade P. B., Valentão P., Gil-Izquierdo A. (2008). Further knowledge on barley (Hordeum vulgare L.) leaves O-glycosyl-C-glycosyl flavones by liquid chromatography-UV diode-array detection-electrospray ionisation mass spectrometry. *Journal of Chromatography A*.

[37] Gao T., Zhang M., Fang Z., Zhong Q. (2017). Optimization of microwave-assisted extraction of flavonoids from young barley leaves. *International Agrophysics*.

[38] Markham K. R., Mitchell K. A. (2003). The mis-identification of the major antioxidant flavonoids in young barley (*Hordeum vulgare*) leaves. *Zeitschrift für Naturforschung C*.

[39] Lee J. H., Park M. J., Ryu H. W. (2016). Elucidation of phenolic antioxidants in barley seedlings (Hordeum vulgare L.) by UPLC-PDA-ESI/MS and screening for their contents at different harvest times. *Journal of Functional Foods*.

[40] Piasecka A., Sawikowska A., Krajewski P., Kachlicki P. (2015). Combined mass spectrometric and chromatographic methods for in-depth analysis of phenolic secondary metabolites in barley leaves. *Journal of Mass Spectrometry*.

[41] Ehrenbergerová J., Březinová Belcredi N., Kopáček J. (2009). Antioxidant enzymes in barley green biomass. *Plant Foods for Human Nutrition*.

[42] Henderson B. (2007). *Cancer-Free: Your Guide to Gentle Non-toxic Healing*.

[43] Barros J., Serrani-Yarce J. C., Chen F., Baxter D., Venables B. J., Dixon R. A. (2016). Role of bifunctional ammonia-lyase in grass cell wall biosynthesis. *Nature Plants*.

[44] Mohan Murali Achary V., Patnaik A. R., Panda B. B. (2012). Oxidative biomarkers in leaf tissue of barley seedlings in response to aluminum stress. *Ecotoxicology and Environmental Safety*.

[45] Wang C.-D., Sun Y., Chen N. (2016). The role of catalase C262T gene polymorphism in the susceptibility and survival of cancers. *Scientific Reports*.

[46] Ferro F. E. D., de Sousa Lima V. B., Soares N. R. M. (2011). Parameters of metabolic syndrome and Its relationship with zincemia and activities of superoxide dismutase and glutathione peroxidase in obese women. *Biological Trace Element Research*.

[47] Kong W., Zhao Y., Liu F., He Y., Tian T., Zhou W. (2012). Fast analysis of superoxide dismutase (SOD) activity in barley leaves using visible and near infrared spectroscopy. *Sensors*.

[48] Song W.-Y., Yang H.-C., Shao H.-B., Zheng A.-Z., Brestic M. (2014). The alleviative effects of salicylic acid on the activities of catalase and superoxide dismutase in malting barley (*Hordeum uhulgare* L.) seedling leaves stressed by heavy metals. *CLEAN - Soil, Air, Water*.

[49] Zhang Y.-X., Hu K.-D., Lv K. (2015). The hydrogen sulfide donor NaHS delays programmed cell death in barley aleurone layers by acting as an antioxidant. *Oxidative Medicine and Cellular Longevity*.

[50] Cisternas P., Lindsay C. B., Salazar P. (2015). The increased potassium intake improves cognitive performance and attenuates histopathological markers in a model of Alzheimer's disease. *Biochimica et Biophysica Acta (BBA) - Molecular Basis of Disease*.

[51] Miura S., Yoshihisa A., Takiguchi M. (2015). Association of hypocalcemia with mortality in hospitalized patients with heart failure and chronic kidney disease. *Journal of Cardiac Failure*.

[52] Zeng Y., Li Y., Yang J. (2017). Therapeutic role of functional components in Alliums for preventive chronic disease in human being. *Evidence-Based Complementary and Alternative Medicine*.

[53] Zeng Y. W., Wang L. X., Du J. (2009). Correlation of mineral elements between milled and brown rice and soils in Yunnan studied by ICP-AES. *Spectroscopy and Spectral Analysis*.

[54] Zeng Y. W., Du J., Yang X. M. (2016). Identification of quantitative trait loci for mineral elements in grains and grass powder of barley. *Genetics and Molecular Research*.

[55] Begley T. P. (2017). Biochemistry: origin of a key player in methane biosynthesis. *Nature*.

[56] Lípová L., Krchnák P., Komenda J., Ilík P. (2010). Heat-induced disassembly and degradation of chlorophyll- containing protein complexes in vivo. *Biochimica et Biophysica Acta (BBA) - Bioenergetics*.

[57] Fedina I., Velitchkova M., Georgieva K., Nedeva D., Çakirlar H. (2009). UV-B response of greening barley seedlings. *Acta Biologica Hungarica*.

[58] Chernomorsky S., Segelman A., Poretz R. D. (1999). Effect of dietary chlorophyll derivatives on mutagenesis and tumor cell growth. *Teratogenesis, Carcinogenesis, and Mutagenesis*.

[59] Zhang H., Zhang N. N., Ma L., Tang J., Tang Y. J. (2014). Optimization of extraction process for chlorophyll from young barley grasses based on response surface methodology. *Food Science*.

[60] on behalf of the HELENA Study group, Mielgo-Ayuso J., Valtueña J. (2017). Fruit and vegetables consumption is associated with higher vitamin intake and blood vitamin status among European adolescents. *European Journal of Clinical Nutrition*.

[61] Lee Y. J., Wang M. Y., Lin M. C., Lin P. T. (2016). Associations between vitamin B-12 status and oxidative stress and inflammation in diabetic vegetarians and omnivores. *Nutrients*.

[62] Dempsey L. A. (2017). Vitamin C for microglia. *Nature Immunology*.

[63] Hoffmann M. R., Senior P. A., Mager D. R. (2015). Vitamin D supplementation and health-related quality of life: a systematic review of the literature. *Journal of the Academy of Nutrition and Dietetics*.

[64] Masri O. A., Chalhoub J. M., Sharara A. I. (2015). Role of vitamins in gastrointestinal diseases. *World Journal of Gastroenterology*.

[65] Brezinová Belcredi N., Ehrenbergerová J., Fiedlerová V., Bĕláková S., Vaculová K. (2010). Antioxidant vitamins in barley green biomass. *Journal of Agricultural and Food Chemistry*.

[66] Zamora-Ros R., Touillaud M., Rothwell J. A., Romieu I., Scalbert A. (2014). Measuring exposure to the polyphenol metabolome in observational epidemiologic studies: current tools and applications and their limits. *American Journal of Clinical Nutrition*.

[67] Tresserra-Rimbau A., Rimm E. B., Medina-Remón A. (2014). Polyphenol intake and mortality risk: a re-analysis of the PREDIMED trial. *BMC Medicine*.

[68] Acar O., Turkan I., Ozdemir F. (2001). Superoxide dismutase and peroxidase activities in drought sensitive and resistant barley (*Hordeum vulgare* L.) varieties. *Acta Physiologiae Plantarum*.

[69] Lee Y.-H., Kim J.-H., Kim S. (2016). Barley sprouts extract attenuates alcoholic fatty liver injury in mice by reducing inflammatory response. *Nutrients*.

[70] Zeng Y., Yang J., Du J. (2015). Strategies of functional foods promote sleep in human being. *Current Signal Transduction Therapy*.

[71] Grandner M. A., Jackson N., Gerstner J. R., Knutson K. L. (2014). Sleep symptoms associated with intake of specific dietary nutrients. *Journal of Sleep Research*.

[72] Yu Y. M., Chang W. C., Chang C. T., Hsieh C. L., Tsai C. E. (2002). Effects of young barley leaf extract and antioxidative vitamins on LDL oxidation and free radical scavenging activities in type 2 diabetes. *Diabetes & Metabolism*.

[73] Yu Y.-M., Wu C.-H., Tseng Y.-H., Tsai C.-E., Chang W.-C. (2002). Antioxidative and hypolipidemic effects of barley leaf essence in a rabbit model of atherosclerosis. *Japanese Journal of Pharmacology*.

[74] Zeng Y., Pu X., Du J., Yang S., Yang T., Jia P. (2012). Use of functional foods for diabetes prevention in China. *African Journal of Pharmacy and Pharmacology*.

[75] Takano A., Kamiya T., Tomozawa H. (2013). Insoluble fiber in young barley leaf suppresses the increment of postprandial blood glucose level by increasing the digesta viscosity. *Evidence-Based Complementary and Alternative Medicine*.

[76] Iyer U. M., Venugopal S. (2010). Management of diabetic dyslipidemia with subatmospheric dehydrated barley grass powder. *International Journal of Green Pharmacy*.

[77] Byun A. R., Chun H., Lee J., Lee S. W., Lee H. S., Shim K. W. (2015). Effects of a dietary supplement with barley sprout extract on blood cholesterol metabolism. *Evidence-based Complementary and Alternative Medicine*.

[78] Seo W. D., Yuk H. J., Curtis-Long M. J. (2013). Effect of the growth stage and cultivar on policosanol profiles of barley sprouts and their adenosine 5′-monophosphate-activated protein kinase activation. *Journal of Agricultural and Food Chemistry*.

[79] Kondo T., Yamamoto K., Kimata A., Ueyama J., Hori Y., Takagi K. (2008). Association of glycemic profiles with whole blood polyamine among middle-aged Japanese men: colorimetric assay using oat and barley seedling polyamine oxidase. *Environmental Health and Preventive Medicine*.

[80] Richter A., Peter E., Pörs Y., Lorenzen S., Grimm B., Czarnecki O. (2010). Rapid dark repression of 5-aminolevulinic acid synthesis in green barley leaves. *Plant and Cell Physiology*.

[81] Kamiyama M., Shibamoto T. (2012). Flavonoids with Potent Antioxidant Activity Found in Young Green Barley Leaves. *Journal of Agricultural and Food Chemistry*.

[82] Zeng Y. W., Du J., Pu X. Y., Yang S. M., Yang T., Jia P. (2011). Strategies of functional food for hypertension prevention in China. *Journal of Medicinal Plants Research*.

[83] Mazzucotelli E., Tartari A., Cattivelli L., Forlani G. (2006). Metabolism of *γ*-aminobutyric acid during cold acclimation and freezing and Its relationship to frost tolerance in barley and wheat. *Journal of Experimental Botany*.

[84] Kim H., Hong H. D., Shin K. S. (2017). Structure elucidation of an immunostimulatory arabinoxylan-type polysaccharide prepared from young barley leaves (*Hordeum vulgare* L.). *Carbohydrate Polymers*.

[85] Moza J., Gujral H. S. (2016). Starch digestibility and bioactivity of high altitude hulless barley. *Food Chemistry*.

[86] Kuge T., Nagoya H., Tryfona T. (2015). Action of an endo-*β*-1,3(4)-glucanase on cellobiosyl unit structure in barley *β*-1,3:1,4-glucan. *Bioscience, Biotechnology, and Biochemistry*.

[87] Kim H., Kwak B. S., Hong H. D., Suh H. J., Shin K. S. (2016). Structural features of immunostimulatory polysaccharide purified from pectinase hydrolysate of barley leaf. *International Journal of Biological Macromolecules*.

[88] Simeonova R., Kondeva-Burdina M., Vitcheva V., Krasteva I., Manov V., Mitcheva M. (2014). Protective effects of the apigenin-O/C-diglucoside saponarin from *Gypsophila trichotoma* on carbone tetrachloride-induced hepatotoxicity in vitro/in vivo in rats. *Phytomedicine*.

[89] Elchuri S., Oberley T. D., Qi W. (2005). CuZnSOD deficiency leads to persistent and widespread oxidative damage and hepatocarcinogenesis later in life. *Oncogene*.

[90] Durham J. J., Ogata J., Nakajima S., Hagiwara Y., Shibamoto T. (1999). Degradation of organophosphorus pesticides in aqueous extracts of young green barley leaves (*Hordeum vulgare* L). *Journal of the Science of Food and Agriculture*.

[91] Schiller M., Hegelund J. N., Pedas P. (2014). Barley metallothioneins differ in ontogenetic pattern and response to metals. *Plant, Cell & Environment*.

[92] Lang U. E., Borgwardt S. (2013). Molecular mechanisms of depression: perspectives on new treatment strategies. *Cellular Physiology and Biochemistry*.

[93] Pytka K., Dziubina A., Młyniec K. (2016). The role of glutamatergic, GABA-ergic, and cholinergic receptors in depression and antidepressant-like effect. *Pharmacological Reports*.

[94] Yamaura K., Shimada M., Fukata H., Nakayama N., Bi Y., Ueno K. (2012). Antidepressant-like effects of young green barley leaf (*Hordeum vulgare* L.) in the mouse forced swimming test. *Pharmacognosy Research*.

[95] Yamaura K., Tanaka R., Bi Y. (2015). Protective effect of young green barley leaf (*Hordeum vulgare* L.) on restraint stress-induced decrease in hippocampal brain-derived neurotrophic factor in mice. *Pharmacognosy Magazine*.

[96] Ohtake H., Yuasa H., Komura C., Miyauchi T., Hagiwara Y., Kubota K. (1985). Studies on the constituents of green juice from young barley leaves: antiulcer activity of fractions from barley juice. *Yakugaku Zasshi*.

[97] Bamba T., Kanauchi O., Andoh A., Fujiyama Y. (2002). A new prebiotic from germinated barley for nutraceutical treatment of ulcerative colitis. *Journal of Gastroenterology and Hepatology*.

[98] Xie W.-H., Wang X.-J., Xiao Y. Y. (2015). Protective effect of selenium-enriched barley seedling on ethanol-induced gastric ulcer in mice. *Journal of Food Science and Biotechnology*.

[99] Kitta K., Hagiwara Y., Shibamoto T. (1992). Antioxidative activity of an isoflavonoid, 2′′-O-glycosylisovitexin isolated from green barley leaves. *Journal of Agricultural and Food Chemistry*.

[100] Kubatka P., Kello M., Kajo K. (2016). Young barley indicates antitumor effects in experimental breast cancer in vivo and in vitro. *Nutrition and Cancer*.

[101] Meng T., Miura C., Irino N., Kondo R. (2015). Evaluation of the production of young green barley plants containing functional ingredients. *American Journal of Plant Sciences*.

[102] Robles-Escajeda E., Lerma D., Nyakeriga A. M., Ross J. A., Kirken R. A. (2013). Searching in mother nature for anti-cancer activity: anti-proliferative and pro-apoptotic effect elicited by green barley on leukemia/lymphoma cells. *PLoS One*.

[103] Meng T.-X., Irino N., Kondo R. (2015). Melanin biosynthesis inhibitory activity of a compound isolated from young green barley (*Hordeum vulgare* L.) in B16 melanoma cells. *Journal of Natural Medicines*.

[104] Zeng Y., Du J., Pu X. (2015). Coevolution between cancer activities and food structure of human being from Southwest China. *BioMed Research International*.

[105] Ferrone M., Raimondo M., Scolapio J. S. (2007). Pancreatic enzyme pharmacotherapy. *Pharmacotherapy*.

[106] Seo K. H., Park M. J., Ra J. E. (2014). Saponarin from barley sprouts inhibits NF-*κ*B and MAPK on LPS-induced RAW 264.7 cells. *Food and Function*.

[107] Cremer L., Herold A., Avram D., Szegli G. (1996). Inhibitory capacity of some fractions isolated from a green barley extract upon TNF alpha production by the cells of the THP-1 human monocytes line. *Roumanian Archives of Microbiology and Immunology*.

[108] Cremer L., Herold A., Avram D., Szegli G. (1998). A purified green barley extract with modulatory properties upon TNF alpha and ROS released by human specialised cells isolated from RA patients. *Roumian Archives of Microbiology and Immunology*.

[109] Gromley J. J. (1995). Green leaves of barley ease arthritis for some. *Better Nutrition for Today’s Living*.

[110] Moon J. K., Shibamoto T. (2009). Antioxidant assays for plant and food components. *Journal of Agricultural and Food Chemistry*.

[111] Templer S. E., Ammon A., Pscheidt D. (2017). Metabolite profiling of barley flag leaves under drought and combined heat and drought stress reveals metabolic QTLs for metabolites associated with antioxidant defense. *Journal of Experimental Botany*.

[112] Lee Y. C., Son J. Y., Kim K. T., Kim S. S. (1994). Antioxidant activity of solvent extract isolated from barley leaves. *Journal of the Korean Society of Food Science and Nutrition*.

[113] Urbonavičiūtė A., Samuolienė G., Brazaitytė A. (2009). The effect of light quality on the antioxidative properties of green barely leaves. *Sodininkystė Ir Daržininkystė*.

[114] Choe J.-H., Jang A., Choi J.-H. (2010). Antioxidant activities of lotus leaves (Nelumbo nucifera) and barley leaves (Hordeum vulgare) extracts. *Food Science and Biotechnology*.

[115] Osawa T., Katsuzaki H., Hagiwara Y., Hagiwara H., Shibamoto T. (1992). A novel antioxidant isolated from young green barley leaves. *Journal of Agricultural and Food Chemistry*.

[116] Choe J.-H., Choi J.-H., Choi Y.-S. (2011). Antioxidant properties of lotus leaf (Nelumbo nucifera) powder and barley leaf (Hordeum vulgare) powder in raw minced pork during chilled storage. *Korean Journal for Food Science of Animal Resources*.

[117] Kang M.-H., Min K.-S., Shibamoto T. (2013). Enhancement of pork quality from pigs fed feeds supplemented with antioxidants containing defatted sesame dregs and dried barley leaves. *International Journal of Nutrition and Food Sciences*.

[118] Sun-zhong M. A. O., Xiao-fang F. A. N., Xiao-mai W. U., Yong-sheng G. O. N. G., Zhe Y. A. N., Liang-gang H. U. (2007). Effects of barley green on serum lipid, MDA, SOD, ET-1 and NO of hyperlipoproteinemia rats. *Food Science*.

[119] Nishiyama T., Hagiwara Y., Hagiwara H., Shibamoto T. (1994). Inhibitory effect of 2^″^-O-glycosyl isovitexin and *α*-tocopherol on genotoxic glyoxal formation in a lipid peroxidation system. *Food and Chemical Toxicology*.

[120] Chen G. T., Zhao L. Y., Qi G. H. (2012). Effect of barley green on nutritional physiological functions of growing rats. *Science and Technology of Food Industry*.

[121] Hokazono H., Omori T., Yamamoto T., Akaoka I., Ono K. (2014). Effects of a fermented barley extract on subjects with slightly high serum uric acid or mild hyperuricemia. *Bioscience, Biotechnology, and Biochemistry*.

[122] Exley E. K., Mielenz T. J., Brady T. J., Xiao C., Currey S. S. (2009). Use of complementary and alternative medicine among patients with arthritis. *Preventing Chronic Disease*.

[123] Yang X. M., Li D., Du J. (2017). Genetic analysis of functional components content in barley grains of RIL population. *Journal of Triticeae Crops*.

[124] Hokazono H., Omori T., Ono K. (2010). Anti-hyperuricemic effect of fermented barley extract is associated with increased urinary uric acid excretion. *Food Science and Technology Research*.

[125] Moussazadeh M., Badamchian M., Hagiwara Y., Hagiwara H., Goldstein A. (1992). Effect of green barley leaf extract (BLE) on human platelets in vitro. *FASEB Journal*.

[126] Liu G., Chen S., Zhong J., Teng K., Yin Y. (2017). Crosstalk between tryptophan metabolism and cardiovascular disease, mechanisms, and therapeutic implications. *Oxidative Medicine and Cellular Longevity*.

[127] Chen T., Li H.-m., Zou D.-L., Du Y.-Z., Shen Y.-H., Li Y. (2014). Preparation of two flavonoid glycosides with unique structures from barley seedlings by membrane separation technology and preparative high-performance liquid chromatography. *Journal of Separation Science*.

[128] Wang X.-J., Yang L.-H., Shi Y.-L., Yang B. (2006). Biologic health effect of barley seedling on mice experiment. *Food Science*.

[129] Xian Y., Zhang L., Song G., Li W.-M., Liao X. (2016). Research progress of nutritional and health function of barley leaves powder. *Food and Nutrition in China*.

[130] Jeon J. R., Choi J. H. (2010). Lactic acid fermentation of germinated barley fiber and proliferative function of colonic epithelial cells in loperamide-induced rats. *Journal of Medicinal Food*.

[131] Duthey B., Hübner A., Diehl S., Boehncke S., Pfeffer J., Boehncke W. H. (2010). Anti-inflammatory effects of the GABA(B) receptor agonist baclofen in allergic contact dermatitis. *Experimental Dermatology*.

[132] Hokazono H., Omori T., Ono K. (2014). Effects of Single and Combined Administration of Fermented Barley Extract and *γ*-Aminobutyric Acid on the Development of Atopic Dermatitis in NC/Nga Mice. *Bioscience, Biotechnology, and Biochemistry*.

[133] Amling M., Barvencik F. (2015). Calcium and vitamin D in osteology. *Zeitschrift für Rheumatologie*.

[134] Choi S. W., Kim S. H., Lee K. S. (2017). Barley seedling extracts inhibit RANKL-induced differentiation, fusion, and maturation of osteoclasts in the early-to-late stages of osteoclastogenesis. *Evidence-Based Complementary and Alternative Medicine*.

[135] Tao E.-F., Yuan T. M. (2016). Vitamin A level and diseases of premature infants. *Chinese Journal of Contemporary Pediatrics*.

[136] Bubko I., Gruber B. M., Anuszewska E. L. (2015). The role of thiamine in neurodegenerative diseases. *Postepy Higieny IMedycyny Doswiadczalnej*.

[137] Mhaidat N. M., Alzoubi K. H., Khabour O. F., Tashtoush N. H., Banihani S. A., Abdul-razzak K. K. (2015). Exploring the effect of vitamin C on sleep deprivation induced memory impairment. *Brain Research Bulletin*.

[138] Wang Q., Prud'homme G., Wan Y. (2015). GABAergic system in the endocrine pancreas: a new target for diabetes treatment. *Diabetes, Metabolic Syndrome and Obesity: Targets and Therapy*.

[139] Waisundara V. Y., Hoon L. Y. (2015). Free radical scavenging ability of Aspalathus Linearis in two in vitro models of diabetes and cancer. *Journal of Traditional and Complementary Medicine*.

[140] Gui L., LaGrange L. P., Larson R. A., Gu M., Zhu J., Chen Q. H. (2012). Role of small conductance calcium-activated potassium channels expressed in PVN in regulating sympathetic nerve activity and arterial blood pressure in rats. *American Journal of Physiology-Regulatory, Integrative and Comparative Physiology*.

[141] Watanabe Y., Kawata K., Watanabe S. (2015). Monitoring technology for gamma-aminobutyric acid productionin polished mochi barley grains using a carbon dioxide sensor. *Journal of Food Science*.

[142] Crowley T., Fitzpatrick J.-M., Kuijper T. (2015). Modulation of TLR3/TLR4 inflammatory signaling by the GABAB receptor agonist baclofen in glia and immune cells: relevance to therapeutic effects in multiple sclerosis. *Frontiers in Cellular Neuroscience*.

[143] Oeltzschner G., Butz M., Baumgarten T. J., Hoogenboom N., Wittsack H. J., Schnitzler A. (2015). Low visual cortex GABA levels in hepatic encephalopathy: links to blood ammonia, critical flicker frequency, and brain osmolytes. *Metabolic Brain Disease*.

[144] Rosa P. B., Neis V. B., Ribeiro C. M., Moretti M., Rodrigues A. L. S. (2016). Antidepressant-like effects of ascorbic acid and ketamine involve modulation of GABA_A_ and GABA_B_ receptors. *Pharmacological Reports*.

[145] Auteri M., Zizzo M. G., Serio R. (2015). GABA and GABA receptors in the gastrointestinal tract: from motility to inflammation. *Pharmacological Research*.

[146] Mangge H., Stelzer I., Reininghaus E. Z., Weghuber D., Postolache T. T., Fuchs D. (2014). Disturbed tryptophan metabolism in cardiovascular disease. *Current Medicinal Chemistry*.

[147] Wu C., Gopal K. V., Moore E. J., Gross G. W. (2014). Antioxidants L-carnitine and D-methionine modulate neuronal activity through GABAergic inhibition. *Journal of Neural Transmission*.

[148] Lin K. H., Lu W. J., Wang S. H. (2014). Characteristics of endogenous *γ*-aminobutyric acid (GABA) in human platelets: functional studies of a novel collagen glycoprotein VI inhibitor. *Journal of Molecular Medicine*.

[149] Sivaranjani N., Rao S. V., Rajeev G. (2013). Role of reactive oxygen species and antioxidants in atopic dermatitis. *Journal of Clinical and Diagnostic Research*.

[150] Liu Q., Wang T., Yu H., Liu B., Jia R. (2014). Interaction between interleukin-1 beta and angiotensin II receptor 1 in hypothalamic paraventricular nucleus contributes to progression of heart failure. *Journal of Interferon & Cytokine Research*.

[151] Huang T. T., Leu D., Zou Y. (2015). Oxidative stress and redox regulation on hippocampal-dependent cognitive functions. *Archives of Biochemistry and Biophysics*.

[152] Zhao W., Li Y., Ma W., Ge Y., Huang Y. (2015). A study on quality components and sleep-promoting effects of GABA black tea. *Food & Function*.

[153] Augier E., Dulman R. S., Damadzic R., Pilling A., Hamilton J. P., Heilig M. (2017). The GABA_B_ positive allosteric modulator ADX71441 attenuates alcohol self-administration and relapse to alcohol seeking in rats. *Neuropsychopharmacology*.

[154] Long Q., Hei Y., Luo Q. (2015). BMSCs transplantation improves cognitive impairment via up-regulation of hippocampal GABAergic system in a rat model of chronic cerebral hypoperfusion. *Neuroscience*.

[155] Seo W. D., Lee J. H., Jia Y., Wu C., Lee S. J. (2015). Saponarin activates AMPK in a calcium-dependent manner and suppresses gluconeogenesis and increases glucose uptake via phosphorylation of CRTC2 and HDAC5. *Bioorganic and Medicinal Chemistry Letters*.

[156] Benedet J. A., Umeda H., Shibamoto T. (2007). Antioxidant activity of flavonoids isolated from young green barley leaves toward biological lipid samples. *Journal of Agricultural and Food Chemistry*.

[157] Mikkelsen B. L., Olsen C. E., Lyngkjær M. F. (2015). Accumulation of secondary metabolites in healthy and diseased barley, grown under future climate levels of CO_2_, ozone and temperature. *Phytochemistry*.

[158] Cruz K. J. C., Oliveira A. R. S. D., Marreiro D. D. N. (2015). Antioxidant role of zinc in diabetes mellitus. *World Journal of Diabetes*.

[159] Jiang X. L., Li M., Zhou J. G., Yang Q. B., Du L. J., Du J. (2011). Plasma paraoxonase-1, oxidized low-density lipoprotein and lipid peroxidation levels in gout patients. *Cell Biochemistry and Biophysics*.

[160] Gómez-Marcos A., Blázquez-Medela A. M., Gamella-Pozuelo L., Recio-Rodriguez J. I., García-Ortiz L., Martínez-Salgado C. (2016). Serum superoxide dismutase is associated with vascular structure and function in hypertensive and diabetic patients. *Oxidative Medicine and Cellular Longevity*.

[161] Kim H., Yun J., Kwon S.-M. (2016). Therapeutic strategies for oxidative stress-related cardiovascular diseases: Removal of excess reactive oxygen species in adult stem cells. *Oxidative Medicine and Cellular Longevity*.

[162] Farajnia S., Meijer J. H., Michel S. (2015). Age-related changes in large-conductance calcium-activated potassium channels in mammalian circadian clock neurons. *Neurobiology of Aging*.

[163] Gokina N. I., Bonev A. D., Phillips J. (2015). Impairment of IKCa channels contributes to uteroplacental endothelial dysfunction in rat diabetic pregnancy. *American Journal of Physiology-Heart and Circulatory Physiology*.

[164] Diness J. G., Bentzen B. H., Sørensen U. S., Grunnet M. (2015). Role of calcium-activated potassium channels in atrial fibrillation pathophysiology and therapy. *Journal of Cardiovascular Pharmacology*.

[165] Jiang Q. (2014). Natural forms of vitamin E: metabolism, antioxidant, and anti-inflammatory activities and their role in disease prevention and therapy. *Free Radical Biology & Medicine*.

[166] van Dalfsen J. H., Markus C. R. (2015). Interaction between 5-HTTLPR genotype and cognitive stress vulnerability on sleep quality: effects of sub-chronic tryptophan administration. *International Journal of Neuropsychopharmacology*.

[167] Mascher M., Gundlach H., Himmelbach A. (2017). A chromosome conformation capture ordered sequence of the barley genome. *Nature*.

